# PfVPS4, an ESCRT AAA-ATPase, is essential for asexual proliferation and gametocyte sexual conversion in *Plasmodium falciparum*

**DOI:** 10.1186/s13071-026-07362-9

**Published:** 2026-04-09

**Authors:** Wenyu Yang, Jing Wu, Yaying Zhou, Zheng Yu, Xiaomin Shang, Jing Huang

**Affiliations:** 1 Human Microbiome and Health Group, Department of Parasitology, Xiangya School of Basic Medical Sciences, Central South University, Changsha, 410013 Hunan China; 2 Hunan Key Laboratory of Immunology and Transmission Control of Schistosomiasis, Yueyang, 414000 Hunan China; 3 Human Microbiome and Health Group, Department of Microbiology, Xiangya School of Basic Medical Sciences, Central South University, Changsha, 410013 Hunan China

**Keywords:** *Plasmodium falciparum*, PfVPS4, ESCRT pathway, Asexual proliferation, Sexual conversion

## Abstract

**Background:**

Malaria, caused by Plasmodium spp., remains a major global health threat. Among them, Plasmodium falciparum is the most pathogenic, and its asexual intraerythrocytic proliferation is the pathological basis. This process has enormous biosynthetic demands and highly relies on the coordinated function of the endomembrane and vesicular transport systems. The transition from asexual proliferation to sexual differentiation similarly involves remodeling of internal membrane complexes, membrane reshaping, and precise protein sorting. In eukaryotic cells, the Endosomal Sorting Complexes Required for Transport (ESCRT) complex is a core machinery for membrane remodeling and endosomal development. However, how the ESCRT system regulates the complex life cycle of Plasmodium, particularly during intraerythrocytic proliferation and sexual conversion, remains an important unresolved question.

**Methods:**

In this study, using Plasmodium falciparum as a model system, we applied CRISPR-Cas9-mediated homologous recombination to achieve conditional knockdown of PfVPS4, the core ATPase of the ESCRT complex — vacuolar protein sorting-associated protein 4 (PfVPS4). Western blotting and immunofluorescence assays were used to assess PfVPS4 abundance and subcellular localization. Tightly synchronized cultures were used to evaluate its effects on parasite growth, merozoite numbers, and gametocyte conversion rate. In vitro protein purification, enzyme kinetics, and site-directed mutagenesis were performed to identify the impact of key residues on PfVPS4 ATPase activity and to validate the synergistic activation by its cofactor PfVta1. In addition, multiple sequence alignment and AlphaFold3 modeling were used to predict and display structural features before and after mutation of key sites.

**Results:**

We successfully generated conditional knockdown lines in both Pf3D7 and PfNF54 parasite strains, enabling effective knockdown at different stages of the intraerythrocytic cycle and during gametocytogenesis. Knockdown of PfVPS4 led to an 84% reduction in asexual progeny parasite numbers, decreased merozoite numbers, and a 46% reduction in gametocyte conversion rate, without affecting subsequent gametocyte maturation. Biochemical assays showed that PfVPS4 ATPase activity is optimal at pH 7.5 and 37°C, and is dependent on Mg²⁺, with a Vmax of 2.23 ± 0.053 U/mg and a Km of 0.086 mM. Site-directed mutagenesis validated the essential role of the canonical catalytic residues (D213, E214) and the species-specific key residues (T161, I288) in maintaining enzymatic activity, and confirmed that the cofactor PfVta1 significantly enhances PfVPS4 activity.

**Conclusion:**

PfVPS4 is essential for normal asexual blood-stage replication and efficient sexual conversion in Plasmodium falciparum. Its knockdown severely disrupts intraerythrocytic proliferative homeostasis and reduces gametocyte conversion, indicating that this protein has a broader role in coordinating parasite proliferation and transmission. Given its essentiality, species‑specific residues, and regulation by PfVta1, PfVPS4 and its complex are attractive antimalarial drug targets.

**Graphical Abstract:**

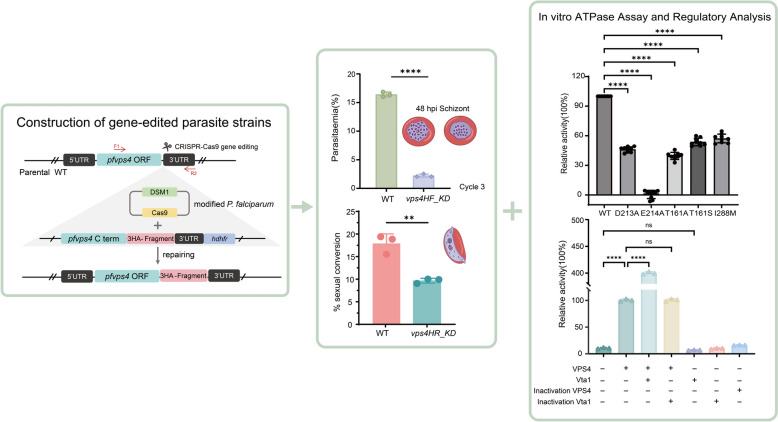

**Supplementary Information:**

The online version contains supplementary material available at 10.1186/s13071-026-07362-9.

## Background

Malaria, a life-threatening disease caused by *Plasmodium* spp., remains a severe global public health challenge [1]. Malaria continues to claim more than half a million lives annually, with the World Health Organization reporting 282 million cases and 610,000 deaths in 2024. The disease disproportionately affects Africa and children under five, imposing a devastating human and socioeconomic cost worldwide [2]. *Plasmodium falciparum*, the most virulent human malaria species, accounts for the majority of this morbidity and mortality. The pathogenesis of malaria is directly linked to the parasite’s intraerythrocytic life cycle, in which merozoites invade red blood cells, undergo asexual replication, and egress to infect new erythrocytes, giving rise to clinical symptoms [3]. In parallel, a subset of parasites differentiates into sexual stages (gametocytes), which are essential for transmission to mosquitoes [4]. Thus, efficient asexual proliferation within erythrocytes and timely transition to gametocytes together underpin both disease severity and onward transmission.

Intraerythrocytic development imposes extreme demands on membrane remodeling and vesicular trafficking. Following invasion, the intraerythrocytic parasite establishes a parasitophorous vacuole and ingests up to ~80% of the host cell cytoplasm via cytostomal endocytosis to fuel rapid growth and replication [5]. A key component of this process is the endocytosis of host hemoglobin through specialized cytostomal structures and its delivery to the digestive food vacuole for degradation via the parasite’s endosomal system [1, 6, 7]. This massive biosynthetic demand necessitates highly efficient endocytosis and membrane trafficking, with cargos ultimately destined for degradative compartments—notably, the food vacuole, which performs lysosome-like functions in *Plasmodium* [1, 6, 8]. In model eukaryotes, such sorting and trafficking processes are primarily governed by the endosomal sorting complex required for transport (ESCRT) complex, Rab GTPases, and SNARE proteins [9–12].

In apicomplexan parasites, however, conserved endosomal machinery may undergo functional “rewiring” that could contribute to the biogenesis of specialized secretory organelles, as well as to the secretion and proper assembly of related secretory proteins, which is crucial for host cell invasion [12–16]. Further, the *Plasmodium* food vacuole (a digestive organelle) exhibits enrichment of the phosphatidylinositol 3-phosphate (PI3P) signal [6], suggesting that related endosomal machinery may also be involved in the transport of nutrients to this compartment. This atypical localization suggests fundamental remodeling of the vesicular transport system, in which endosomal components are repurposed for nutrient acquisition and metabolic compartmentalization [1, 6]. Functional studies reinforce this notion of pathway reorganization. PfRab5a has no function in endocytosis, and PfSand1 inactivation does not impair endocytic uptake in trophozoites [1, 17, 18], indicating divergence from canonical roles. In contrast, PfVPS45, PfRbsn5L, and PfRab5b are essential for endocytosis, suggesting that cytostome function connects into the endosomal transport pathway [1, 6]. These observations suggest that *P. falciparum* optimizes its compact genome by assigning stage- and pathway-specific functions to individual trafficking proteins, yet the molecular machinery that coordinates endocytic trafficking, particularly the endosomal sorting system, remains incompletely defined.

The ESCRT machinery constitutes a central regulatory mechanism of endosomal trafficking in eukaryotic cells. In model systems, ESCRT-0, ESCRT-I, ESCRT-II, and ESCRT-III complexes assemble sequentially to mediate cargo selection, membrane invagination, and membrane scission, thereby driving the formation of intraluminal vesicles within multivesicular bodies (MVBs) [11, 19–21]. The AAA-ATPase VPS4, assisted by its cofactor Vta1, acts as the “recycling engine” of this pathway by disassembling ESCRT-III polymers from membranes and regenerating a pool of free subunits for subsequent rounds of membrane remodeling [22–24]. Beyond MVB biogenesis, ESCRT-III and VPS4 participate in a wide range of membrane remodeling events, including viral budding, plasma membrane repair, and cytokinetic abscission [22–24]. Intriguingly, malaria parasites lack the canonical ESCRT-0, ESCRT-I, and ESCRT-II subcomplexes [25], yet retain ESCRT-III together with apparent homologs of VPS4 and Vta1, implying that they operate a streamlined but potentially indispensable ESCRT machinery. An earlier study found that PfVPS4 was located in the cytoplasm of the malaria parasite and suggested that it might be involved in the formation process of MVB-like structures [26].

Despite this, the roles of ESCRT components in *P. falciparum* remain poorly understood. ESCRT-III has been implicated in the biogenesis of extracellular vesicles (EVs) originating from the parasitophorous vacuole (PV) lumen and in protein export during infection [27], suggesting that ESCRT-mediated membrane remodeling contributes to host–parasite communication. However, whether and how PfVPS4 regulates key life-cycle processes in *P. falciparum*, including both asexual proliferation and sexual development, is not yet understood. Likewise, the existence and function of a *P. falciparum* Vta1 (PfVta1) cofactor, its ability to regulate PfVPS4 ATPase activity, and the biochemical features of PfVPS4 itself have not been systematically characterized. This knowledge gap limits our understanding of how ESCRT pathways are adapted in apicomplexan parasites and constrains the evaluation of PfVPS4 as a potential intervention target.

To address these questions, we conducted a functional analysis of PfVPS4 in *P. falciparum* that combines in vivo phenotyping with in vitro enzymology. We systematically evaluated PfVPS4 function during blood-stage development using conditional knockdown. Our results demonstrate its essential role in intraerythrocytic schizogony and sexual conversion. Through biochemical reconstitution, we defined the enzymatic properties of PfVPS4 and identified its key catalytic residues. We also identified PfVta1 as a regulatory cofactor that activates PfVPS4 ATPase activity. Together, these findings establish the PfVPS4-PfVta1 complex as a critical ESCRT effector in malaria parasites and provide a molecular framework for understanding how ESCRT-based membrane remodeling supports *P. falciparum* blood-stage development.

## Methods

### Parasite culture

*P. falciparum* strains 3D7 and NF54 were maintained in human O + erythrocytes at 2% hematocrit in complete RPMI 1640 medium supplemented with 0.5% Albumax II, 0.2% sodium bicarbonate, 25 mM HEPES, and 50 μg/mL hypoxanthine. Cultures were incubated at 37 °C in a gas mixture (5% O_2_, 5% CO_2_, 90% N_2_) with medium changes every 48 h [28]. Ring-stage parasites were synchronized using 5% sorbitol, and late schizonts were enriched by Percoll sorbitol density gradient centrifugation.

### Plasmid construction

The clustered regularly interspaced short palindromic repeats (CRISPR)/Cas9-based plasmids pL6CS-v*ps4*-*ha*-*ddfkbp* and pL6CS-*vps4*-*ha-glmS* were constructed as described previously [29]. Briefly, sgRNAs targeting the vps4 locus were cloned into the pL6CS vector between XhoI and AvrII sites. Homology arms (~0.5 kb) were amplified from genomic DNA, and HA-tag sequences were incorporated by overlap extension polymerase chain reaction (PCR). The resulting fragments were inserted into the AflII and PstI sites of pL6CS. All constructs were verified by Sanger sequencing. Primer sequences are listed in Table S1.

### Generation of transgenic parasite lines

For parasite transfection, 100 μg of pL6CS-v*ps4*-*ha*-*ddfkbp* or pL6CS-*vps4*-*ha*-*glmS* plasmid was co-transfected with 100 μg of pUF1-Cas9 plasmid (in a total volume of 150 μl ddH_2_O) into fresh erythrocytes by electroporation. Percoll-enriched schizonts were used for transfection: those of the 3D7 strain for the pL6CS-*vps4-ha-ddfkbp* plasmid, selected with WR99210 and DSM1; and those of the NF54 strain for the pL6CS*-vps4-ha-glmS* plasmid, selected with WR99210 and BSD (Cat# R21001). Transfected parasites for both lines were subjected to drug selection for 3–4 weeks until parasitemia became microscopically detectable. Correct genomic integration was confirmed by PCR and sequencing. Monoclonal lines were obtained by limited dilution. Verification primers are presented in Table S1.

### Growth curve analysis

Wild-type (WT) and *vps4HF_KD* (PfVPS4‑HA‑ddFKBP) parasites were cultured in vitro. Following strict synchronization, the developmental window was restricted to 6 h. At the ring stage, cultures were adjusted to an initial parasitemia of 0.1% and divided into two groups, maintained in the presence or absence of 0.5 μM Shld. Cultures were maintained for four intraerythrocytic cycles, and parasitemia was monitored at each trophozoite stage by counting Giemsa-stained thin blood smears. Growth curves were generated and analyzed using GraphPad Prism 10.1.2.

### Gametocyte induction and analysis

To minimize the potential indirect impact of PfVPS4 defects during the intraerythrocytic stages on gametocyte conversion rates—and thereby focus on its direct role—we implemented a rigorous multistep experimental strategy. The procedure was as follows:

First, the conditional knockdown line *vps4HR_KD* (PfVPS4‑HA‑glmS) parasites were synchronized to ~5 h ring stages, evenly seeded into 6‑well plates at an initial parasitemia of 2%, and cultured in complete RPMI 1640 medium. When parasitemia exceeded 10%, this timepoint was designated as day 0 (parasitemia 1). At this point, 5 mM glucosamine (GlcN) were added to the experimental group to induce PfVPS4 knockdown. Within the subsequent 12–15 h, both the experimental and control groups were treated with 50 mM N‑acetyl‑glucosamine (GlcNAc; A3286, Sigma-Aldrich) for four consecutive days to eliminate the existing asexual parasite population. During this period (days 2 and 4), microscopic examination confirmed the absence of new viable rings, ensuring effective suppression of asexual proliferation.

The core objective of this design was to remove the late‑stage schizonts that might otherwise be reduced due to PfVPS4 impairment, thereby diminishing the potential indirect influence of asexual‑stage defects on subsequent gametocyte output. This allowed for the analysis to focus more directly on the effect of gene knockdown on the gametocyte‑conversion event itself. Gametocytemia (parasitemia 2) was determined by morphological identification of gametocytes in Giemsa-stained smears. The sexual conversion rate was calculated using the formula: sexual conversion rate (%) = parasitemia 2/parasitemia 1. Each experiment included three biological replicates.

### Fluorescence microscopy

Immunofluorescence assays were performed as previously described [30] to determine the subcellular localization of PfVPS4. Parasites were tightly synchronized and collected at the ring, trophozoite and schizont stages. After lysis of infected erythrocytes with 0.15% saponin, parasites were immediately fixed on ice in ice-cold 4% paraformaldehyde. Parasites were incubated with rabbit anti-HA primary antibody (1:500; HUABIO, HA721750) followed by iFluor™ 488-conjugated goat anti-rabbit immunoglobulin (Ig)G secondary antibody (1:500; HUABIO, HA1121), each for 1 h at room temperature. Between incubations, slides were washed three times with phosphate-buffered saline (PBS). Nuclei were stained with 4′,6-diamidino-2-phenylindole (DAPI) (Beyotime) according to the manufacturer’s instructions. Fluorescence imaging was performed on a STELLARIS 5 confocal microscope (Leica Microsystems) using a 100 × oil immersion objective. Images were processed in ImageJ.

### Western blotting

Western blotting was performed as described previously [31], with minor modifications. Briefly, infected erythrocytes were lysed in 0.15% saponin, and parasite pellets were resuspended in an equal volume of 2 × sodium dodecyl sulfate (SDS) loading buffer and boiled at 100 °C for 5 min. Proteins were separated on 10% or 12% SDS‑polyacrylamide gel electrophoresis (PAGE) gels, depending on the expected molecular weights of PfVPS4‑HA‑ddFKBP (~64 kDa) and PfVPS4‑HA‑glmS (~54 kDa), and transferred to PVDF membranes. Membranes were probed with rabbit anti‑HA (1:5,000, Cat# HA721750, HUABIO), mouse anti‑histone H3 (1:5,000, Cat# ET1601‑30, HUABIO), and mouse anti‑β-actin (1:5,000, Cat# 66009-1-Ig, Proteintech) antibodies, followed by incubation with HRP‑conjugated goat anti‑mouse or anti‑rabbit IgG (1:8,000, Cat# HA1006 and HA1001, HUABIO). Signals were detected by ECL and imaged using a ChemiDoc^™^ XRS + system (Bio‑Rad) with Image Lab™ Software.

### Sequence analysis and three-dimensional structure prediction

Nucleotide and amino acid sequence information was retrieved from PlasmoDB (https://plasmodb.org/plasmo/app/). The deduced amino acid sequence was analyzed by ExPASy Proteomics Server (http://www.expasy.ch/tools/). Multiple sequence alignments were generated with Clustal Omega (http://www.ebi.ac.uk/Tools/msa/clustalo/) and visualized using ESpript v.3.0 server [32]. Three-dimensional structures were predicted with AlphaFold3.0, which is widely regarded as a reliable protein structure prediction platform [33]. Structural images were prepared using PyMOL [34]

### Gene cloning, protein expression, purification, and identification of PfVPS4 and PfVta1 in *E. coli*

The coding sequences of *pfvps4* (PF3D7_1457500) and *pfvta1* (PF3D7_0103000) were amplified by PCR using gene-specific primers (S1 Table). PCR products and the pET28a (+) vector were digested with BamHI and Hind III and ligated by T4 DNA ligase (Takara) with a molar ratio of 4:1 for insert to vector. Recombinant plasmids were transformed into *E. coli* DH5α and selected on LB agar plates containing 50 μg/mL kanamycin. True positive clones were screened by colony PCR and verified by Sanger sequencing before storage at −80 °C.

Recombinant plasmids encoding PfVPS4 and PfVta1 were isolated from *E. coli* DH5α and expressed in *E. coli* Rosetta (DE3) as described previously [35], with minor modifications. Briefly, proteins were purified from soluble fractions by Ni^2^^+^-affinity chromatography followed by size-exclusion chromatography Superdex 200 16/600 column (GE, USA) under native conditions. Protein purity was assessed by 12% SDS-PAGE, and concentration was determined by Bradford assay.

### ATPase activity and kinetic analysis of PfVPS4

ATP hydrolysis by PfVPS4 was monitored by a malachite-green-based colorimetric assay using a commercial ATPase assay kit (Nanjing Jiancheng Bioengineering) to measure released inorganic phosphate under conditions [36]. Standard reactions contained 100 mM Tris–HCl (pH 7.4), 20 mM KCl, 6 mM MgCl_2_, and defined amounts of PfVPS4. Initial rates were measured across a series of ATP concentrations and fitted to the Michaelis–Menten equation using GraphPad Prism to obtain *Kₘ* and *Vₘₐₓ* values. The malachite green reagent was prepared as described and stabilized at 4 °C before use [37]. To assess environmental effects, 0.5 mg/mL PfVPS4 was incubated in buffers of varying pH, temperature, or metal ion composition. Reactions were initiated with 1.2 mM ATP, incubated for 10 min at 37 °C, and terminated with 32% sodium citrate. Absorbance was measured at 620 nm after 15 min. All values were normalized to the maximum activity within each experiment. All assays were performed in triplicate.

### Site-specific amino acid mutation and expression purification of PfVPS4

Site-specific mutations (e.g., E214A, D213A, T161A, T161S, I288M) were introduced into the *pfvps4* coding sequence using complementary mutagenic primers (Supplementary Table S1). All oligonucleotides were synthesized by Sangon Biotech (Shanghai) and designed with flanking restriction sites for cloning into linearized pET-28a (+). Mutant constructs were generated by two-step overlap extension PCR. Fragments containing the desired mutation were first amplified with mutation-specific and terminal primers, then used as templates for full-length assembly. The resulting constructs were transformed into *E. coli* DH5α, and positive clones were selected and verified. Mutant proteins were expressed and purified following the same procedures as the WT-PfVPS4.

### ATPase activity of PfVPS4 mutants and PfVta1-stimulated PfVPS4 activity

ATPase activity of PfVPS4 mutants was determined using a malachite-green-based assay described above. Mutant proteins were quantified by bicinchoninic acid (BCA) assay and adjusted to 0.5 mg/mL in optimized reaction buffer. Reactions were initiated by adding 10 μL of 1.2 mM ATP in 96-well plates and incubating at 37 °C for 10 min. Subsequently, 80 μL malachite green reagent and 10 μL 32% sodium citrate were added to stop the reaction. After mixing and incubation at 37 °C for 15 min, absorbance at 620 nm was recorded.

For PfVta1 stimulation assays, PfVPS4 (0.5 mg/mL) was preincubated with PfVta1 (1.2 mg/mL) or serial dilutions thereof for 5 min at room temperature before ATP addition. Relative activity was calculated as a percentage of the maximum activity within each experiment. The Vps4 activity was statistically analyzed using GraphPad Prism 10.1.2 software for PfVta1 (both inactivated and non-inactivated) stimulation. The activity was standardized on the basis of the level of ATP hydrolysis by PfVPS4. The ATP enzyme activity assay was conducted according to the previous method.

## Results

### PfVPS4 deficiency disrupts steady-state proliferation of *P. falciparum*-infected erythrocytes

**Figure 1:**
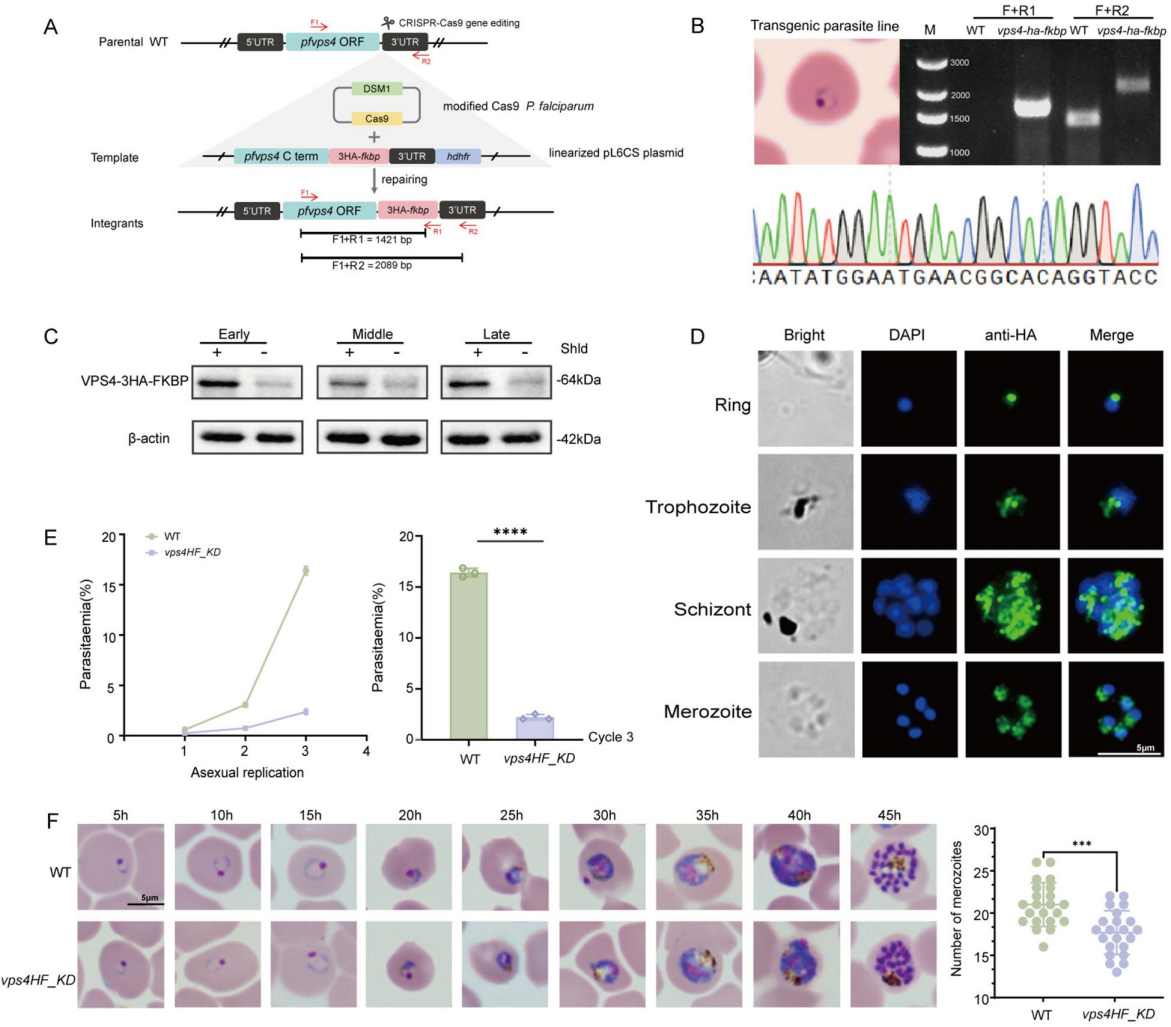
Konckdown of PfVPS4 disrupts proliferative homeostasis during the intraerythrocytic phase of *P. falciparum*. **A** Schematic of the conditional knockdown strategy showing integration of the ddFKBP destabilization domain at the 3' end of the PfVPS4 genomic locus. **B** PCR verification of correct genomic integration in the transgenic parasite line. **C** Western blot analysis of PfVPS4-HA expression across ring, trophozoite, and schizont stages in the presence or absence of Shld. Lysates were probed with anti-HA and anti-β-actin antibodies; molecular weights (kDa) are indicated. Data are representative of three biological replicates. Quantitative analysis is shown in Supplementary Fig. S2A. **D** Immunofluorescence images of intraerythrocytic stages and free merozoites probed with anti-HA antibody (green) and DAPI (blue), showing 3 × HA-tagged VPS4 expression and localization. Scale bar: 5 μm. **E** Growth kinetics of PfVPS4 knockdown parasites cultured with or without Shld. Synchronized parasites were monitored over three replication cycles using Giemsa-stained smears. The bar graph (right) compares parasite densities at the end of cycle 3. Data represent mean ± SD from three biological replicates, each with three technical replicates (****P < 0.0001). **F** Representative Giemsa staining images of the morphology and growth of the insect body at 5 h intervals when the ring-shaped stage began to be knocked down were sampled at the designated time points. Scale bar: 5 μm. **G** Quantification of nuclei per schizont at 46 h post-invasion in Shld-treated and untreated parasites. Data are from three independent experiments, error bars indicate SD (***P < 0.0001)

The *P. falciparum* VPS4 homolog (PF3D7_1457500) encodes a 1260 bp open reading frame corresponding to a ~48 kDa protein. To investigate its function, we generated a conditional knockdown parasite line (*Pfvps4HF_KD*) by inserting the ddFKBP destabilizing domain at the 3' terminus of the endogenous *vps4* locus using the CRISPR/Cas9 Gene editing system [38] (Fig. 1A). Transfected parasites were selected under WR99210 and DSM1 pressure for approximately 3 weeks until parasites became microscopically detectable. Correct genomic integration was confirmed by PCR using combinations of gene-specific and insert-specific primers (F1 + R1 and F1 + R2), which yielded amplicons specific to the transgenic line (Fig. 1B). Monoclonal *Pfvps4HF_KD* line was subsequently obtained by limiting dilution.

In this system, the PfVPS4-HA-FKBP protein is stabilized by the Shield-1(Shld) ligand and degraded upon Shld withdrawal. To assess knockdown efficiency, we examined PfVPS4 expression in ring, trophozoite, and schizont stages at 48 h post-treatment in the presence (WT) or absence (*Pfvps4HF_KD*) of Shld by Western blotting. Quantitative analysis revealed that Shld withdrawal led to a marked reduction in PfVPS4 levels: protein abundance decreased by 86.31% in ring stages, 55.05% in trophozoites, and 76.73% in schizonts compared with Shld-treated controls (Fig. 1C and Supplementary Fig. S2A).

Indirect immunofluorescence assay using an anti-HA antibody showed that PfVPS4 is widely expressed during the asexual blood stages, localizing to both the perinuclear region and the cytoplasm (Fig. 1D). This localization pattern is consistent with a previous study, which reported that PfVPS4 is present not only in the cytoplasm, but also in structures such as the endoplasmic reticulum and the digestive vacuole [26]. The agreement between these studies underscores its broad subcellular distribution and suggests dynamic associations with multiple membrane compartments.

To evaluate the impact of PfVPS4 knockdown on erythrocytic development, tightly synchronized parasites (6 h window) were cultured for three consecutive cycles with or without Shld. Giemsa-stained thin smears were examined each cycle to monitor morphology and parasitemia. PfVPS4 knockdown severely impaired asexual proliferation, resulting in a cumulative reduction in parasite density and an 84% ± 0.2 decrease in parasitemia by the third cycle compared with controls (Fig. 1E). All drug treatment experiments included rigorous parallel growth controls to confirm that Shld alone did not contribute to the observed phenotype (Supplementary Fig. S1A). These data establish that PfVPS4 is essential for maintaining proliferative capacity during the blood stages.

To pinpoint the developmental defect, we analyzed intraerythrocytic progression following PfVPS4 knockdown. The majority of parasites (70–80%) exhibited no developmental delay or morphological abnormalities in knockdown versus control parasites. However, PfVPS4 knockdown caused a clear reduction in the number of merozoites per mature schizont at 44–46 h post-invasion (Fig. 1F), indicating that PfVPS4 contributes to normal schizogony and merozoite formation. Interestingly, after one complete replication cycle, the proportion of ring-stage parasites decreased by 56.8 ± 0.16% compared with the control. By the third cycle, this decline was further amplified to approximately 84%. This progressive and severe reduction in parasitemia exceeds what would be expected from the decrease in merozoite numbers alone. This discrepancy could reflect that, although a subset of merozoites possess countable nuclei, they may be functionally inactive or that additional defects exist in subsequent steps such as merozoite egress or erythrocyte invasion. These two possibilities are not mutually exclusive and both warrant further investigation.

### PfVPS4 deficiency disrupts the homeostasis of parasite sexual conversion

**Figure 2:**
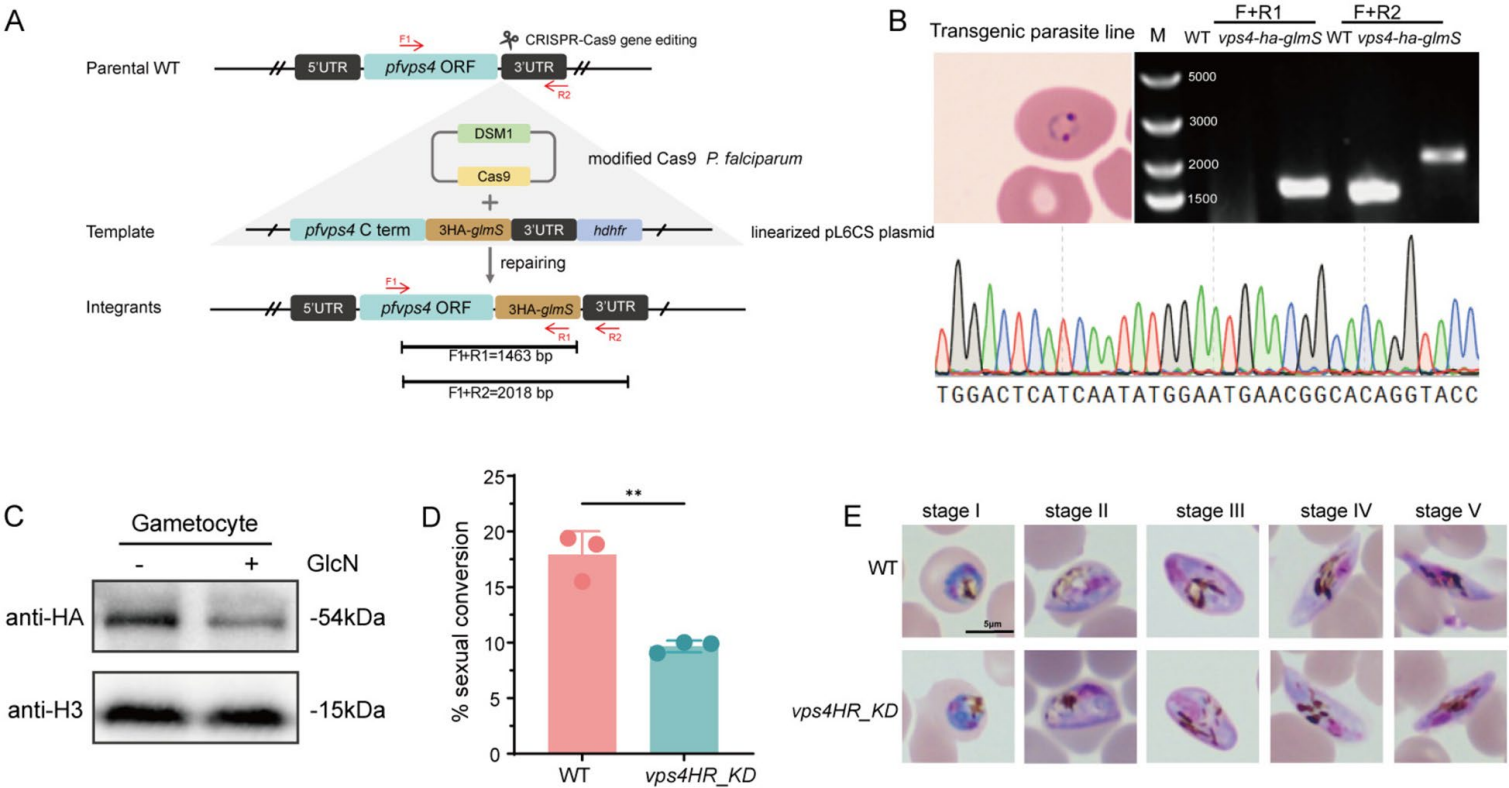
PfVPS4 deficiency reduces gametophyte conversion rate but does not affect maturation. **A** Strategy for conditional PfVPS4 knockdown in gametocytes using glmS ribozyme insertion at the 3’ end of the genomic locus. **B** PCR validation of glmS integration into the PfVPS4 locus. **C** Western blot analysis of PfVPS4-HA expression in mature gametocytes cultured with or without 5 mM glucosamine (GlcN). Membranes were probed with anti-HA and anti-H3 antibodies. Quantitative analysis is shown in Supplementary Fig. S2B. **D** Gametocyte conversion rates in the presence or absence of GlcN. Knockdown caused a reduction in conversion efficiency under identical gametocyte induction conditions. Data are from three independent experiments, error bars indicate SD (**P < 0.0028). **E** Schematic representation of gametocyte development stages under GlcN-treated and untreated conditions. **F** Giemsa-stained images of gametocytes from the second developmental cycle following PfVPS4 knockdown. Scale bar: 5 μm. Morphological maturation was not affected by PfVPS4 knockdown.

To investigate the role of PfVPS4 in gametocytogenesis, we generated a conditional knockdown line in the NF54 strain by inserting a glucosamine (GlcN)-inducible glmS ribozyme at the 3' end of the PfVPS4 coding sequence (Fig. 2A) [39]. Transgenic parasites were selected under drug pressure, and correct genomic integration was verified by PCR with gene-specific and insert-specific primers (Fig. 2B). Monoclonal *Pfvps4HR_KD* lines were obtained by limiting dilution.

To assess the impact of PfVPS4 knockdown on sexual commitment and development, we induced gametocytogenesis in WT and *Pfvps4HR_KD* parasite lines and monitored developmental progression via morphological analysis of Giemsa-stained thin blood smears. In these experiments, we minimized interference from potential asexual replication defects as much as possible. First, control experiments confirmed that glucosamine (GlcN) treatment alone did not affect gametocyte conversion rates (Supplementary Fig. S1B). Subsequently, Western blot analysis verified that GlcN induction led to a significant reduction (54.8%) in PfVPS4 protein levels (Fig. 2C and Supplementary Fig. S2B). Phenotypic analysis revealed that, compared with the untreated WT group, the gametocyte conversion efficiency of *pfvps4HR_KD* was decreased by approximately 46% (Fig. 2D). Notably, although conversion efficiency was impaired, the *Pfvps4HR_KD* gametocytes that formed exhibited normal morphology (Fig. 2E) and matured at a rate comparable to that of WT parasites.

These results suggest that, under conditions designed to minimize interference from the asexual stages, PfVPS4 may be important for efficient sexual conversion, while appearing largely dispensable for the subsequent maturation of gametocytes.

### Expression, purification, and enzymatic characterization of PfVPS4

**Figure 3:**
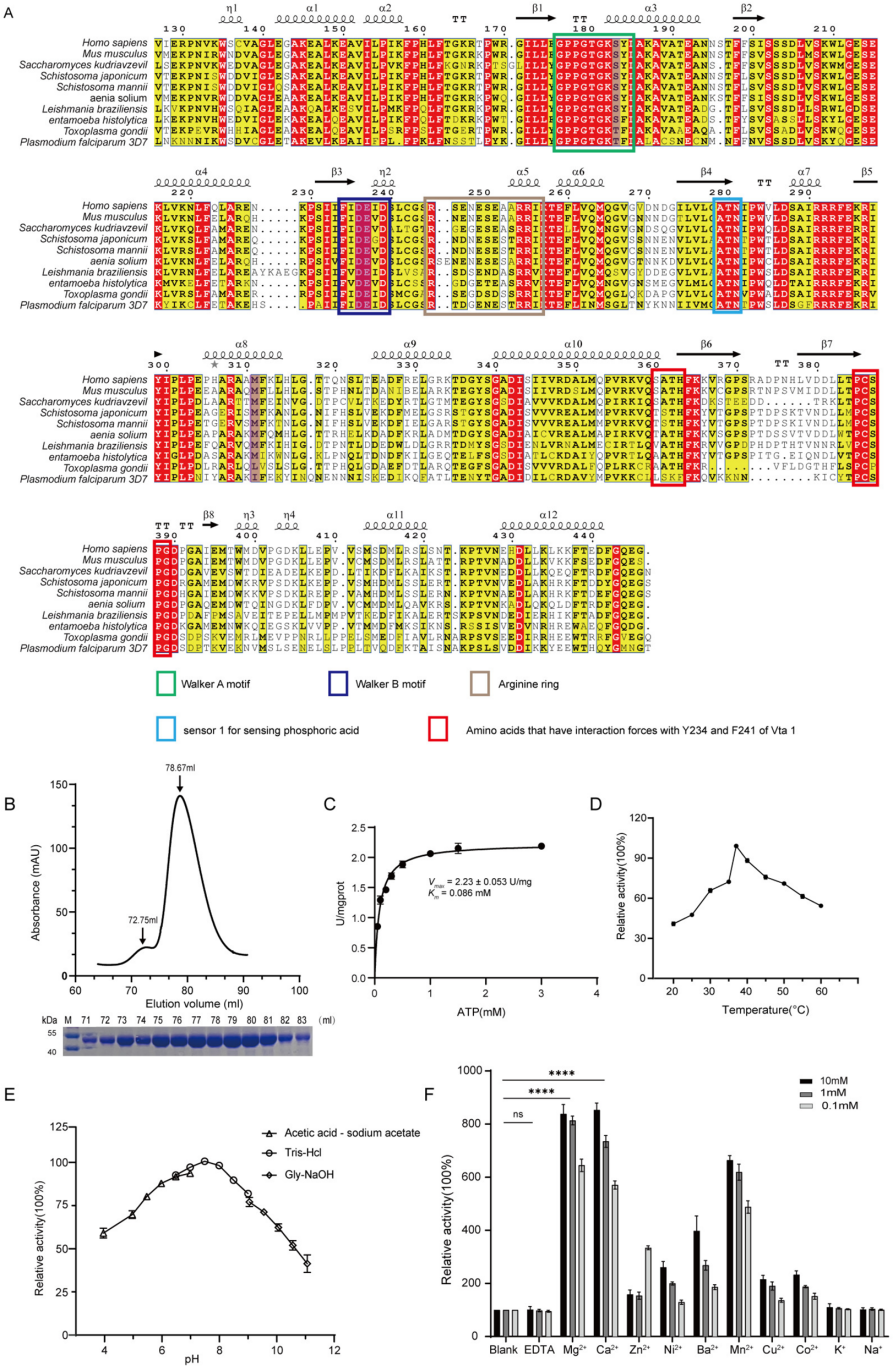
Sequence analysis of PfVPS4 and enzymatic characterization after protein purification. **A** Multiple sequence alignment of VPS4 homologs from various species. Conserved domains and functional motifs are indicated. The amino acids for site-directed mutagenesis are separately marked by purple shadows. **B** Size-exclusion chromatography profile of purified PfVPS4 (Elution Volume: 72.75 and 76.78 ml). Inset: SDS-PAGE analysis of the purified protein. The original gel image is provided in Supplementary Fig. S3. **C** Steady-state enzyme kinetics of PfVPS4 under optimized conditions (pH 7.5, 37 °C, Mg^2^^+^). Kinetic parameters were determined as *V*_max_ = 2.23 ± 0.053 U/mg and *K*_m_ = 0.086 ± 0.010 mM. **D** Temperature profile of PfVPS4 ATPase activity. Activity at 37 °C was set as 100%. Data represent mean ± SD from three independent experiments. **E** pH dependence of PfVPS4 activity across different buffer systems. Activity at pH 7.5 was normalized to 100%. **F** Metal ion requirement for PfVPS4 activity. Activity in the absence of metal ions was set as 100%. Data are presented as mean ± SD; (****P < 0.0001) compared with the no-metal control.

To elucidate the enzymatic mechanism of PfVPS4, we first performed multiple sequence alignment of its AAA-ATPase domain (Fig. 3A), focusing on the conservation of key functional motifs. Although the overall sequence of PfVPS4 is relatively divergent from other VPS4 eukaryotic homologs, specific amino acid substitutions were observed even within core motifs such as Walker A, Walker B, and the Vta1-interaction loop, which are essential for adenosine triphosphate (ATP) binding, hydrolysis, and cofactor interaction [40]. These observations suggested potential species-specific features in PfVPS4 catalysis, prompting us to express and purify recombinant PfVPS4 for biochemical analysis.

The full-length *Pfvps4* coding sequence was cloned into pET-28a (+) to generate a recombinant construct with an N-terminal 6 × His tag. Following expression in *E. coli* Rosetta (DE3) stains and IPTG induction, PfVPS4 was produced predominantly in soluble form. The protein was purified by Ni^2^^+^-NTA affinity chromatography followed by gel filtration chromatography. SDS-PAGE confirmed an apparent molecular mass of ~48 kDa, consistent with the predicted size, and the result of size-exclusion chromatography, by comparison with the standard curve, showed that it may be monomers and dimers in solution (Fig. 3B). Grayscale scanning indicated a final purity exceeding 95%, suitable for subsequent biochemical assays.

We then established a malachite-green-based ATPase activity assay to quantify inorganic phosphate release during ATP hydrolysis and to define the kinetic properties of PfVPS4. Under optimized reaction conditions, initial rates measured across a range of ATP concentrations followed typical Michaelis–Menten kinetics, yielding a *K*_*m*_ of 0.086 mM and a *V*_max_ of 2.23 ± 0.053 U/mg protein (Fig. 3C). Temperature-dependence analysis revealed maximal activity at 37 °C, with more than 50% activity retained between 37 °C and 60 °C but substantially reduced activity at 20 °C (Fig. 3D). The pH-dependence analysis performed using three different buffer systems (sodium acetate, Tris–HCl, and glycine–NaOH) showed that the activity reached its maximum at pH 7.5 (Fig. 3E). The activity of PfVPS4 remained highly stable within the pH range of 7.0 to 8.5, and tends to retain higher activity in moderately acidic compared with alkaline condition. In metal ion dependency assays, PfVPS4 activity strictly depends on divalent metal ions; Mg^2^^+^ and Ca^2^^+^ were the most effective, enhancing activity by 6–8-fold relative to controls (Fig. 3F). Together, these data establish PfVPS4 as a classical AAA-ATPase with strict divalent cation dependence and optimal activity under physiological-like conditions, providing a biochemical framework for understanding its catalytic function in parasite biology.

### Analysis of key functional site mutations and ATPase catalytic mechanisms of PfVPS4

**Figure 4:**
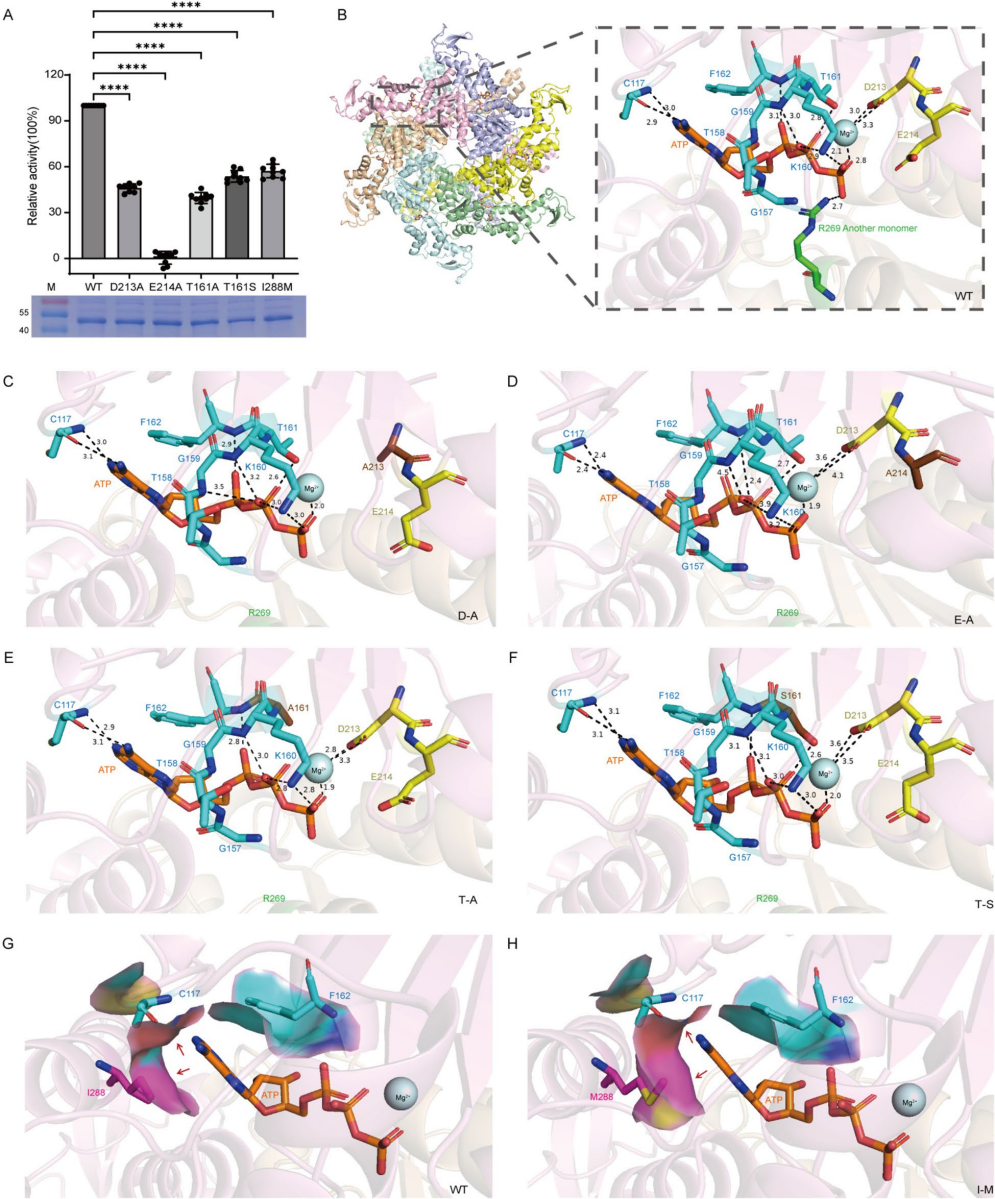
Point-mutation analysis and predicted structural modeling of PfVPS4. **A** Purified point-mutant proteins (SDS-PAGE shown below) and their corresponding activity comparisons with the wild-type (WT) group. Original blots and gels are shown in Supplementary Fig. S4. **B**–**H** Predicted three-dimensional structural models illustrating the spatial hydrogen bond network before and after mutation, generated computationally using AlphaFold3.0. Potential force-generating positions are indicated by black bond lines, and mutated amino acids are labeled in the lower right corner. In these predicted structural models, ATP-binding residues are colored blue, ATP-hydrolyzing residues yellow, adenine-stacking residues magenta, and mutated residues dark brown.

Sequence alignment revealed amino acid variations within the ATP-binding motifs (e.g., Walker A), the conserved catalytic core, and the adenine-binding loop. To investigate the functional significance of these nonconserved positions, we generated a series of point mutations at these key residues (T161A, T161S, D213A, E214A, and I288M) (Fig. 4A). We further predicted residues involved in hydrogen bonding with ATP within the minimal dimeric unit and modeled their spatial arrangement and ligand-binding environment. Comparative analysis with human and other eukaryotic VPS4 homologs revealed several distinctive substitutions at key positions involved in ATP binding and hydrolysis. Serine is replaced by threonine at position 161 (S161T), tyrosine is replaced by phenylalanine at position 162 (Y162F), and methionine at position 288 is not conserved among apicomplexans, being replaced by isoleucine in Plasmodium (M288I) and leucine in Toxoplasma (M288L), whereas other core catalytic residues remain highly conserved. (Fig. 4B).

Mutations in the catalytic residues (D213A, E21AQ) caused a severe or complete loss of ATPase activity, confirming their essential roles in ATP hydrolysis. The highly conserved acidic residue (E214) within the Walker B motif likely functions as the catalytic base that activates the nucleophilic water [41, 42]. In engineered constructs, The E214A mutation allows ATP (or its analog ATPγS) binding while strongly impairing hydrolysis. Similarly, D213 is predicted to participate in Mg^2^^+^ coordination and transition-state stabilization, and its substitution to alanine also sharply reduced activity (Fig. 4D). Notably, T161A caused a more pronounced loss of activity than T161S (Fig. 4E, F). Although threonine and serine share similar chemical properties, The proximity of T161 to the Mg^2^^+^ coordination network suggests that its side-chain hydroxyl may contribute to stabilizing water molecules or forming hydrogen bonds with phosphate groups. Replacement with alanine removes this hydroxyl, disrupting critical interactions, whereas substitution with serine partially preserves function.

Mutation of the adenine-stacking residue I288 to methionine (I288M) also led to a decrease in enzyme activity. Efficient ATP hydrolysis requires precise stacking of the adenine ring to stabilize the ATP-bound conformation and correctly orient the γ-phosphate for nucleophilic attack [43].

We propose that the substitutions at F162 and I288 in the wild-type protein represent critical evolutionary adaptations. Y162 and M288 are both susceptible to reactive oxygen species (ROS), which can lead to protein inactivation. The M/I–Y/F substitutions are therefore not neutral; replacing oxidation-prone methionine with inert, purely hydrophobic isoleucine constitutes a refined adaptive strategy. This eliminates a potential oxidative damage hotspot, thereby preserving the functional integrity of this essential enzyme within the hostile oxidative environment of the host erythrocyte. Furthermore, the sulfur atom of Cys117 likely participates in weak van der Waals contacts or C–H···S interactions, which help stabilize the assembly, compensate for the potential loss of hydrogen bonding due to the Y162F substitution, and create a highly complementary interface for ATP binding.

Taken together, our mutagenesis and AlphaFold3 structural analyses reveal that efficient PfVPS4 catalysis depends on both direct catalytic residues (e.g., E214) and a substrate-stabilizing microenvironment (e.g., T161 and M288). Perturbation of any element in this network leads to diminished or abolished ATPase activity.

### Expression and purification of PfVta1 and its stimulation of PfVPS4 ATPase activity

**Figure 5:**
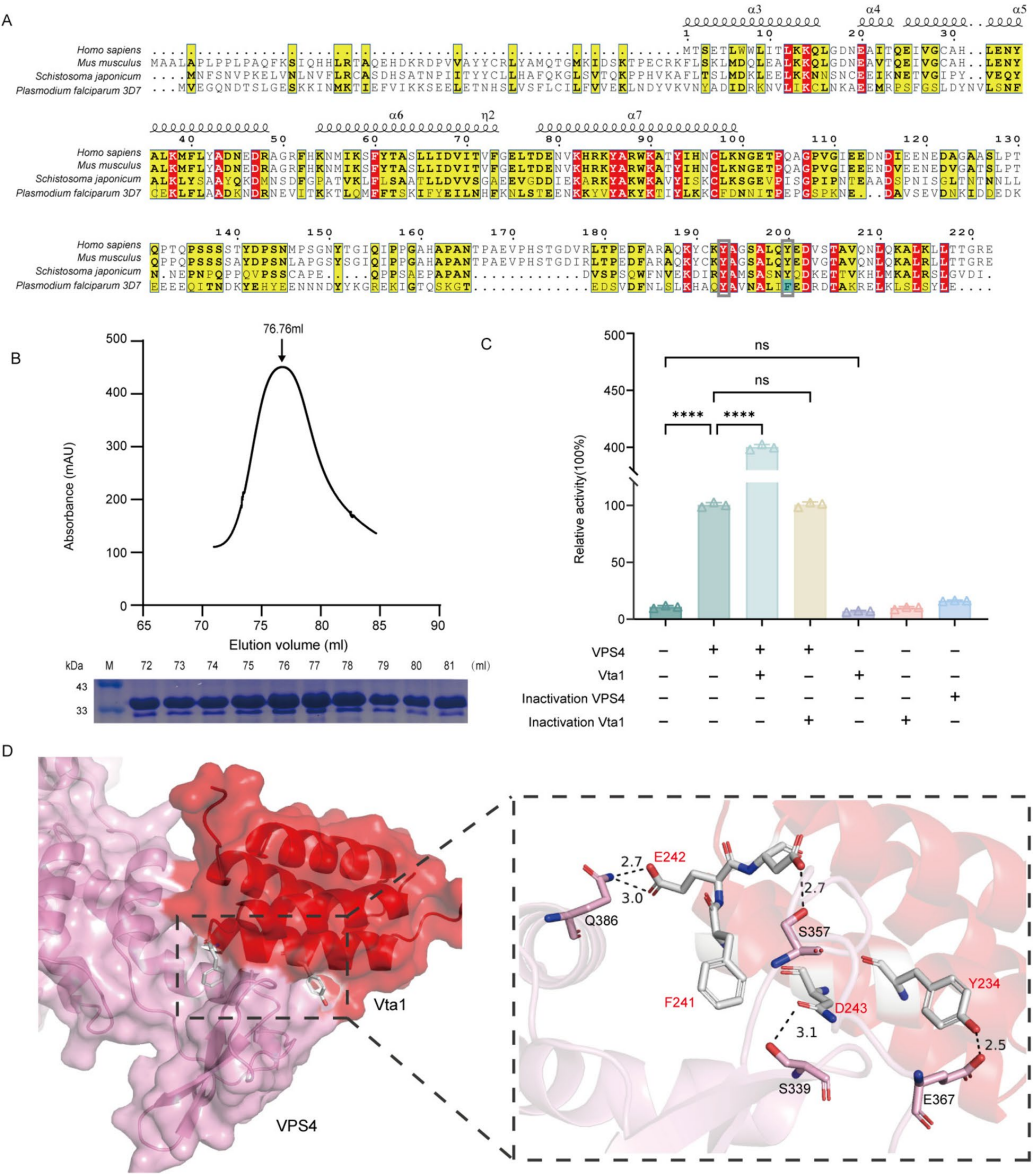
Purification of PfVta1 and its stimulatory effect on PfVPS4, with a predicted schematic of the structural binding interface. **A** Multiple sequence alignment of Vta1 homologs from various species. Domains and active sites are labeled as indicated. The silver box marks two important amino acids that interact with PfVPS4, and the blue shadow indicates that mutations have occurred. **B** Gel filtration chromatography profile of purified PfVta1. The protein eluted at the 76.76 ml peak. SDS-PAGE analysis of the eluted fractions from the Superdex 200 16/600 column is shown below. Original blots and gels are provided in Supplementary Fig. S3. **C** Validation of PfVta1-mediated stimulation of PfVPS4 activity. “+” indicates the presence of the l bnhuiabeled (treated) protein on the left; “−” indicates no addition. **D** Predicted structural models of the PfVta1–PfVPS4 interaction interface. Detailed illustration of the interaction interface, with potential force-generating positions indicated by black bond lines.

In eukaryotes, the VPS4 function is commonly modulated by auxiliary proteins such as Vta1. To determine whether a similar regulatory mechanism exists in *P. falciparum*, we first performed sequence alignment using PlasmoDB, which identified a conserved Vta1 homolog in the parasite genome. Basic Local Alignment Search Tool (BLAST) analysis revealed a putative PfVta1 coding sequence with conserved domains and species-specific variations (Fig. 5A). The full-length PfVta1 sequence was cloned into pET-28a (+), generating an N-terminal 6 × His-tagged construct. Following IPTG induction in *E. coli* Rosetta (DE3), PfVta1 was expressed in soluble form and purified to high homogeneity by Ni^2^^+^-NTA affinity chromatography followed by gel filtration. The SDS-PAGE analysis showed that its apparent molecular weight was approximately 31 kDa, which was consistent with the theoretical prediction. By comparing with the standard curve, the results of size exclusion chromatography indicated that it might exist in a dimer form in the solution (Fig. 5B).

Current crystallographic and structural studies have demonstrated that VPS4 assembles into an active hexamer (or dodecamer) upon substrate binding, while the dimeric form of Vta1 can bridge adjacent VPS4 protein subunits, which may help promote and stabilize the active multimeric assembly of VPS4 [42, 44, 45]. To determine whether PfVta1 acts as such an activity-enhancing cofactor, we employed the malachite green ATPase assay and found that it significantly enhanced the ATP hydrolysis rate of Vps4 (Fig. 5C), which firmly establishing PfVta1 as a positive regulator of PfVPS4. To explore the structural basis of this regulation, we analyzed predicted structural models of the PfVPS4-PfVta1 complex (Fig. 5D). Predicted structural analysis revealed that, in *Plasmodium*, one of a pair of tyrosine residues (Fig. 5A)—critical for PfVPS4–PfVta1 interaction and Vta1 dimerization in other eukaryotes—is substituted with phenylalanine (F241). Although F241 does not contribute hydrogen bonds, this substitution does not markedly alter the overall binding interface with PfVPS4. This observation is consistent with our sequence alignment and structural comparison (Fig. 3A and S5), which revealed that despite additional mutations at the Vta1-VPS4 interaction interface, the overall binding architecture remains largely conserved. Because tyrosine is more susceptible to oxidative damage due to its phenolic hydroxyl groups, whereas phenylalanine contains an inert benzene ring, this substitution may reduce oxidative sensitivity at the protein–protein interface without compromising structural integrity. We speculate that such a conservative adaptation helps maintain PfVPS4–PfVta1 complex stability under host-derived oxidative stress.

In summary, we identified and functionally validated PfVta1 as a critical cofactor of PfVPS4 in *P. falciparum*. Combining biochemical assays with predicted structural models of the interaction interface, we demonstrate that PfVta1 strongly enhances PfVPS4 ATPase activity and note the presence of species-specific sequence variations in their binding interface, providing a basis for further mechanistic studies of ESCRT pathway regulation in malaria parasites.

## Discussion

In this study, we systematically delineated the critical role of PfVPS4 in the blood-stage development of *P. falciparum*. Through conditional knockdown in different parasite strains, we demonstrated that PfVPS4 is essential for maintaining asexual proliferation and gametocyte commitment. PfVPS4 deficiency led to impaired asexual proliferation, as evidenced by reduced parasitemia and fewer merozoites per schizont. Indirect immunofluorescence assay showed that PfVPS4 is widely expressed during the asexual blood stages, localizing to both the perinuclear region and the cytoplasm. Given that a small proportion of PfVPS4 has also been reported in association with the endoplasmic reticulum, nucleus, and other compartments [26], this broad distribution pattern leads us to speculate that it may participate in conserved ESCRT-III-related processes in *Plasmodium*, such as maintaining nuclear envelope integrity [11, 46, 47] and endoplasmic reticulum autophagy (ER-phagy) [48, 49].

It is important to note that the canonical multicomponent ESCRT pathway is not fully conserved in *Plasmodium*; most of its members are absent. Although evidence suggests that other ESCRT‑III components retained in the parasite may still participate in conserved processes such as extracellular vesicle formation [27], this raises a deeper question: does PfVPS4 retain the conserved function—seen in the ESCRT machinery of other eukaryotes—of participating in membrane fission and remodeling [11, 50, 51], or has it undergone functional specialization in *Plasmodium*? Could it even exhibit both characteristics? For example, in *Plasmodium*, the HOPS/CORVET complex, which belongs to the same VPS protein family and is closely associated with endosomal trafficking, maintains specific endocytic functions by transporting hemoglobin to the digestive vacuole and delivering invasion‑related proteins [12]. This hints that VPS4 may likewise assume a specialized membrane‑trafficking role in the parasite. Notably, the extent of parasitemia reduction following PfVPS4 knockdown exceeds what can be explained merely by a decrease in merozoite numbers, implying that its functional impairment may extend beyond schizont maturation. It has been reported that a GFP‑PfVps4 mutant localizes to endosomal structures that intersect with the rhoptry biogenesis pathway [26]. Given the growing body of evidence indicating that rhoptries are formed along the endocytic route [12–16], we speculate that PfVPS4 might influence parasite proliferation and invasion by participating in the normal development and trafficking of the endosomal system. Therefore, future studies should further explore whether and how VPS4 is integrated into these parasite‑specific biological processes. This specific hypothesis awaits validation through subsequent experiments.

Our analysis indicates that PfVPS4 knockdown reduces gametocyte conversion efficiency but does not impair subsequent maturation. Although our experimental design aimed to minimize interference from asexual-stage defects, the precise mechanistic step by which PfVPS4 affects conversion remains unclear. Given the broader roles of the ESCRT machinery in exosome biogenesis and signal transduction in other eukaryotes [11, 52], we hypothesize that PfVPS4 may regulate conversion by participating in vesicular release pathways or host–parasite interactions. A plausible model, therefore, is that its function is more critical during early differentiation events rather than in later maturation—a hypothesis that awaits direct experimental validation.

Biochemical characterization confirmed that PfVPS4 is a canonical AAA-ATPase, with ATP hydrolysis strictly dependent on Mg^2^^+^ and optimal activity under physiological temperature and pH. The enzyme’s broad activity range under varying conditions may reflect an evolutionary adaptation that enables it to maintain essential function despite fluctuations in the host environment. Catalytic residues D213 and E214, together with species-specific positions T161 and I288, are essential for maintaining enzyme activity. This conserved eukaryotic regulatory mechanism is maintained in *P. falciparum*, where PfVta1 functions as a positive regulatory cofactor to enhance PfVPS4 activity.

Although this study highlights the essential role of PfVPS4 in asexual proliferation and gametocyte commitment, several questions remain. We have not yet generated complete PfVPS4 knockout lines, thus the absolute requirement of PfVPS4 for blood-stage survival needs further validation. Moreover, the precise mechanisms by which PfVPS4 maintains proliferative homeostasis, including its effects on nutrient uptake, organelle biogenesis, and invasion processes, require detailed characterization through molecular markers and subcellular localization studies. Additionally, the underlying mechanisms contributing to the severe intraerythrocytic phenotypic decline remain incompletely understood. In addition to the quantifiable defects in parasite proliferation described above, we also noted that some ring‑stage parasites lacking PfVPS4 exhibited bright, clear vacuoles. A comprehensive quantification and mechanistic characterization will be pursued in follow-up work.

## Conclusion

PfVPS4 is essential for normal asexual blood-stage replication and efficient sexual conversion in Plasmodium falciparum. Its knockdown severely disrupts intraerythrocytic proliferative homeostasis and reduces gametocyte conversion, indicating that this protein has a broader role in coordinating parasite proliferation and transmission. Given its essentiality, the species-specificity of its key residues, and its regulation by the positive modulator PfVta1, PfVPS4 and its complex with PfVta1 are attractive targets for future antimalarial drug development.

## Supplementary Information

Supplementary Material 1: S1 Table. Primers used for assessing correct genomic integration of plasmids. Figure S1 Control experiments demonstrate no effect of Shld-1(A) and GlcN(B) on wild-type parasite growth. A: Wild-type parasites were cultured for four complete intraerythrocytic cycles in the presence or absence of Shld (0.5 μM). Parasitaemia was monitored at the trophozoite stage of each cycle by counting parasites on Giemsa-stained thin blood smears. No significant difference in parasite proliferation was observed between Shld-treated and untreated cultures (*P* > 0.05), confirming that Shld itself does not affect parasite growth under these conditions. B: For gametocyte induction experiments, parasites were cultured in the presence or absence of GlcN (5 mM), and gametocyte conversion rates were enumerated. No significant difference in gametocyte conversion was observed between GlcN-treated and untreated cultures (*P* > 0.05), confirming that GlcN itself does not affect gametocyte conversion under these conditions (Supplementary Fig. S1). Figure S2 Quantitative analysis results of Western blot. A: Western blot quantification demonstrates successful PfVPS4 knockdown in ring, trophozoite, and schizont stages following Shld-1 withdrawal. Protein levels were normalized to β-actin loading control and expressed relative to Shld-treated controls (set as 100%). B: Western blot quantification demonstrates successful knockdown of PfVPS4 following 5mM GlcN treatment during gametocyte induction. Data represent mean ± SD from three biological replicates. The consistent reduction in PfVPS4 levels across all developmental stages confirms the robustness of the knockdown system. Figure S3 Purification analysis of recombinant PfVPS4 and PfVta1. A: SDS-PAGE analysis of PfVPS4 protein following purification by gel filtration chromatography. B: SDS-PAGE analysis of PfVta1 protein after gel filtration purification. The target proteins were specifically expressed and exhibited > 95% purity as determined by grayscale scanning. Figure S4 Purification analysis of recombinant PfVPS4 mutants. A: SDS-PAGE analysis of five PfVPS4 point mutants following initial purification by Ni-NTA affinity chromatography. B: SDS-PAGE verification of the purified mutants after additional gel filtration chromatography. All protein samples were normalized to uniform concentration by BCA assay prior to electrophoresis. Figure S5 Structural analysis of the PfVta1–PfVPS4 interaction interface based on predicted models. Predicted surface representation of key residues at the PfVta1–PfVPS4 binding interface (see Fig. 3A). PfVta1 is shown in red (residues Y234, F241 highlighted) and PfVPS4 in pink. The computationally predicted surface models reveal a complementary binding interface between the two proteins.

## Data Availability

No datasets were generated or analyzed during the current study.

## Abbreviations

ESCRT: Endosomal sorting complex required for transport
VPS4: Vacuolar protein sorting-associated protein 4
PI3P: Phosphatidylinositol 3-phosphate
MVBs: Multivesicular bodies
PV: Parasitophorous vacuole
EVs: Extracellular vesicles
WT: Wild-type
GlcNAc: N-acetyl-D-glucosamine
GlcN: Glucosamine
ER: Endoplasmic reticulum
