# Supplementary material for: PfVPS4, an ESCRT AAA-ATPase, is essential for asexual proliferation and gametocyte sexual conversion in Plasmodium falciparum
Source: Parasit Vectors. 2026 Apr 9;19:219. doi: 10.1186/s13071-026-07362-9 (PMC13185166; doi:10.1186/s13071-026-07362-9)
Supplement: Supplementary file 1 — Supplementary Material 1: S1 Table. Primers used for assessing correct genomic integration of plasmids. Figure S1 Control experiments demonstrate no effect of Shld-1(A) and GlcN(B) on wild-type parasite growth. A: Wild-type parasites were cultured for four complete intraerythrocytic cycles in the presence or absence of Shld (0.5 μM). Parasitaemia was monitored at the trophozoite stage of each cycle by counting parasites on Giemsa-stained thin blood smears. No significant difference in parasite proliferation was observed between Shld-treated and untreated cultures (P > 0.05), confirming that Shld itself does not affect parasite growth under these conditions. B: For gametocyte induction experiments, parasites were cultured in the presence or absence of GlcN (5 mM), and gametocyte conversion rates were enumerated. No significant difference in gametocyte conversion was observed between GlcN-treated and untreated cultures (P > 0.05), confirming that GlcN itself does not affect gametocyte conversion under these conditions (Supplementary Fig. S1). Figure S2 Quantitative analysis results of Western blot. A: Western blot quantification demonstrates successful PfVPS4 knockdown in ring, trophozoite, and schizont stages following Shld-1 withdrawal. Protein levels were normalized to β-actin loading control and expressed relative to Shld-treated controls (set as 100%). B: Western blot quantification demonstrates successful knockdown of PfVPS4 following 5mM GlcN treatment during gametocyte induction. Data represent mean ± SD from three biological replicates. The consistent reduction in PfVPS4 levels across all developmental stages confirms the robustness of the knockdown system. Figure S3 Purification analysis of recombinant PfVPS4 and PfVta1. A: SDS-PAGE analysis of PfVPS4 protein following purification by gel filtration chromatography. B: SDS-PAGE analysis of PfVta1 protein after gel filtration purification. The target proteins were specifically expressed and exhibited > 95% pur [file 13071_2026_7362_MOESM1_ESM.pdf]

Supplementary Table

Primers of pL6CS-*vps4-ha-ddfkbp*

GGAAGTATATATGACAAGAGCC	F1
ATATAGTCAGTTTGGCTCTCTG	R1
TTTTTTACAAAATGCTTAAGGATAATTTAAAACTTTTCACACTCT	R2

Primers of pL6CS-*vps4-ha-glms*

GGAAGTATATATGACAAGAGCC	F1
TTTCTCTTTGTTCAAGGAGTC	R1
TTTTTTACAAAATGCTTAAGGATAATTTAAAACTTTTCACACTCT	R2

Primer for PfVPS4 protein molecule cloning and site-directed mutagenesis protein

CGCGGATCCATGGACTCTGAAGAAACAATAA	VPS4-F1
CCCAAGCTTTTATGTACCGTTCATTCCATAT	VPS4-R2
CTTTATTGCAGAAATTGATTCCTTATGTGGATCAAGAACTGAC	D213A F2
GGAATCAATTTCTGCAATAAAG	D213A R1
CAATAATCTTTATTGATGCAATTGATTCCTTATGTGGATCAAG	E214-A F2
CAATTGCATCAATAAAGATTATTGC	E214A R1
CTGGTACAGGGAAAGCATTCCTTGCATTAGCTTGTTCGAATG	T161A F2
GAATGCTTTCCCTGTACCAGGTGG	T161A R1
CTGGTACAGGGAAATCGTTCCTTGCATTAGCTTGTTCGAATG	T161S F2
GAACGATTTCCCTGTACCAGGTGG	T161S R1
CAAAAATGTTTGAAAAATATATCAATCAAAATG	I288M F2
GATATATTTTTCAAACATTTTTGCTCTTGC	I288M R1

Primers for cloning the PfVta1 protein molecule

GGAATTCCATATGATGGTTGAAGGGCAAAATG	Vta1-F
CCCAAGCTTTTATTCTAAATATGAAAGGG	Vta1-R

Supplementary Figure

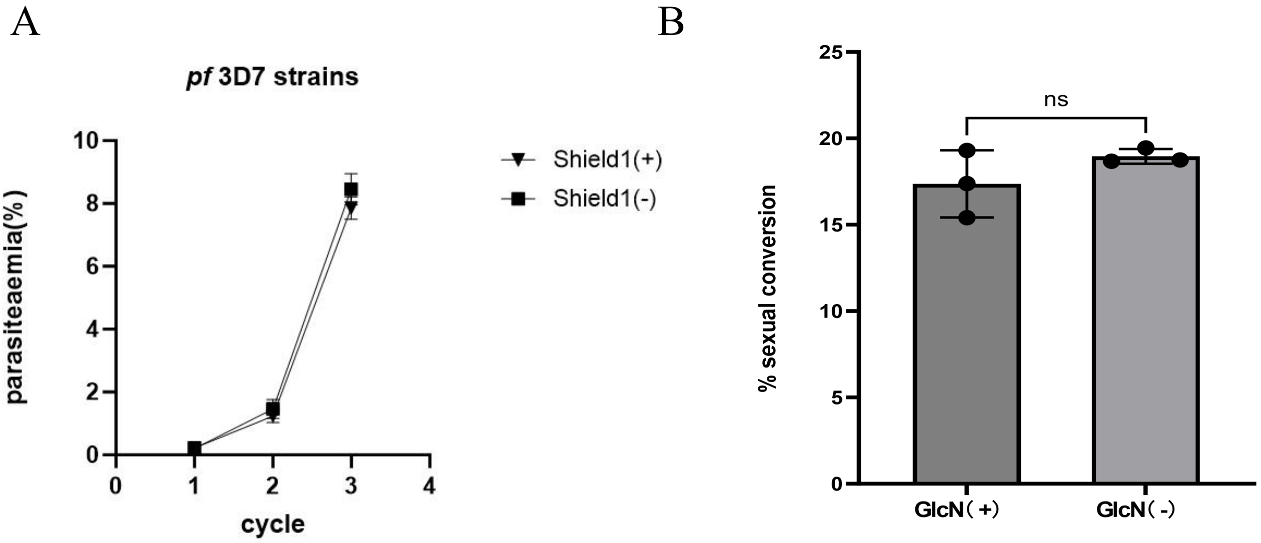

Supplementary Figure S1. Control experiments demonstrate no effect of Shield-1(A) and GlcN(B) on wild-type parasite growth. Wild-type parasites were cultured for four complete intraerythrocytic cycles in the presence or absence of Shld and GlcN. Parasitaemia was monitored at the trophozoite stage of each cycle by counting parasites on Giemsa-stained thin blood smears. No significant difference in parasite proliferation was observed between Shld-treated and untreated cultures ($p > 0.05$), confirming that Shld itself does not affect parasite growth under these conditions

A B

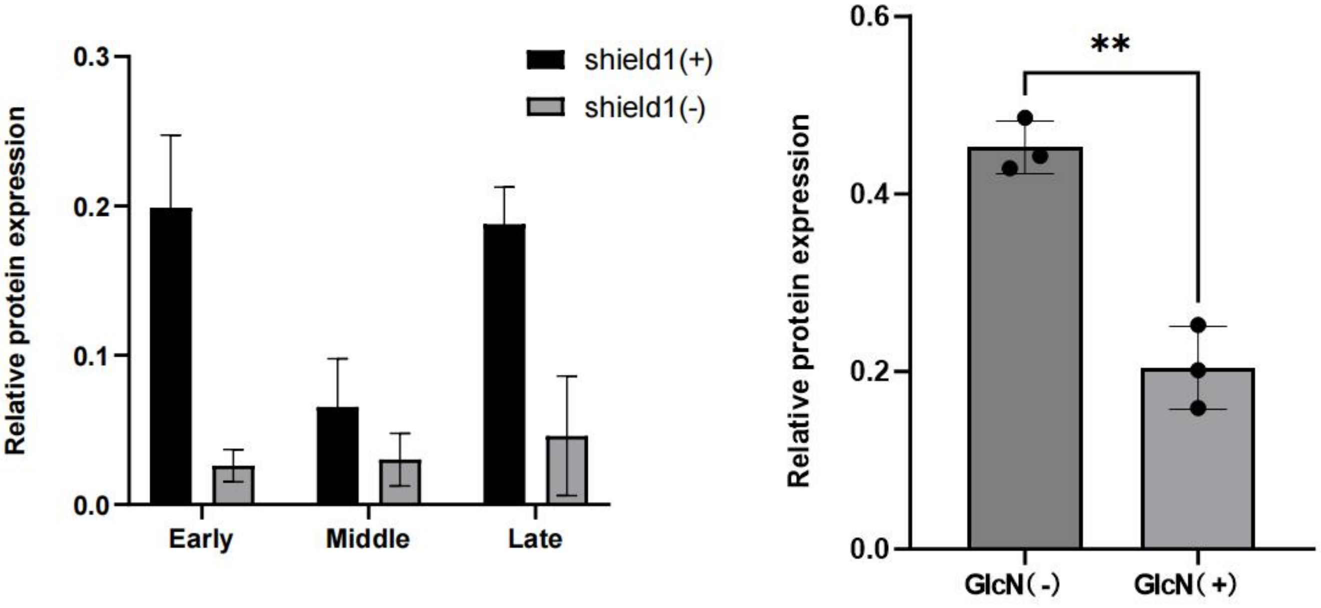

Supplementary Figure S2. Efficient PfVPS4 depletion. A: Western blot quantification demonstrates successful PfVPS4 knockdown in ring, trophozoite, and schizont stages following Shield-1 withdrawal. Protein levels were normalized to β-actin loading control and expressed relative to Shld-treated controls (set as 100%). **B:** Western blot quantification demonstrates successful knockdown of PfVPS4 following GlcN treatment during gametocyte induction. Data represent mean ± SD from three biological replicates. The consistent reduction in PfVPS4 levels across all developmental stages confirms the robustness of the knockdown system.

A B

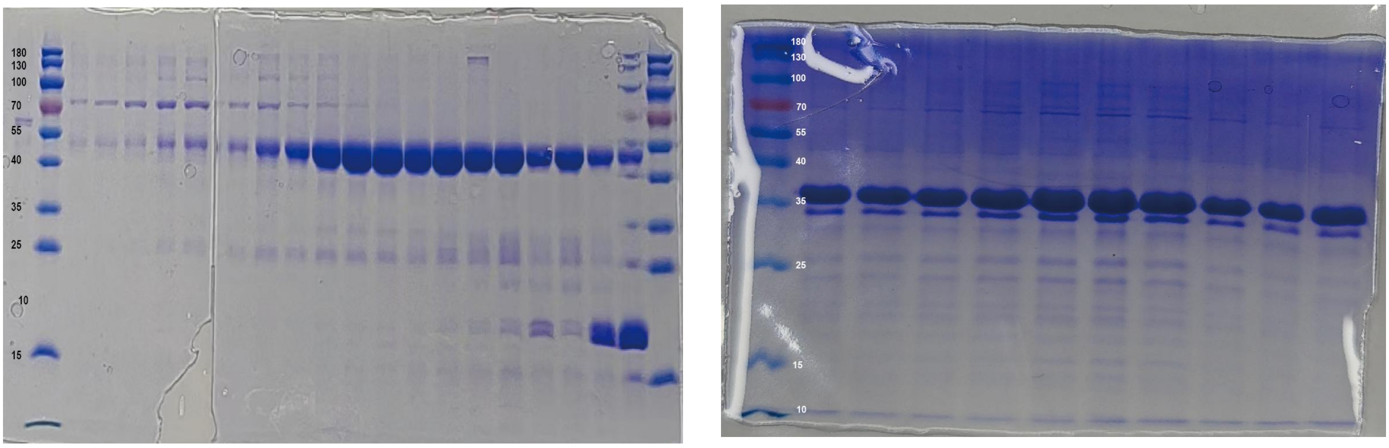

Supplementary Figure S3. Purification analysis of recombinant PfVPS4 and PfVta1

A: SDS-PAGE analysis of PfVPS4 protein following purification by gel filtration chromatography. **B:** SDS-PAGE analysis of PfVta1 protein after gel filtration purification. The target proteins were specifically expressed and exhibited >95% purity as determined by grayscale scanning.

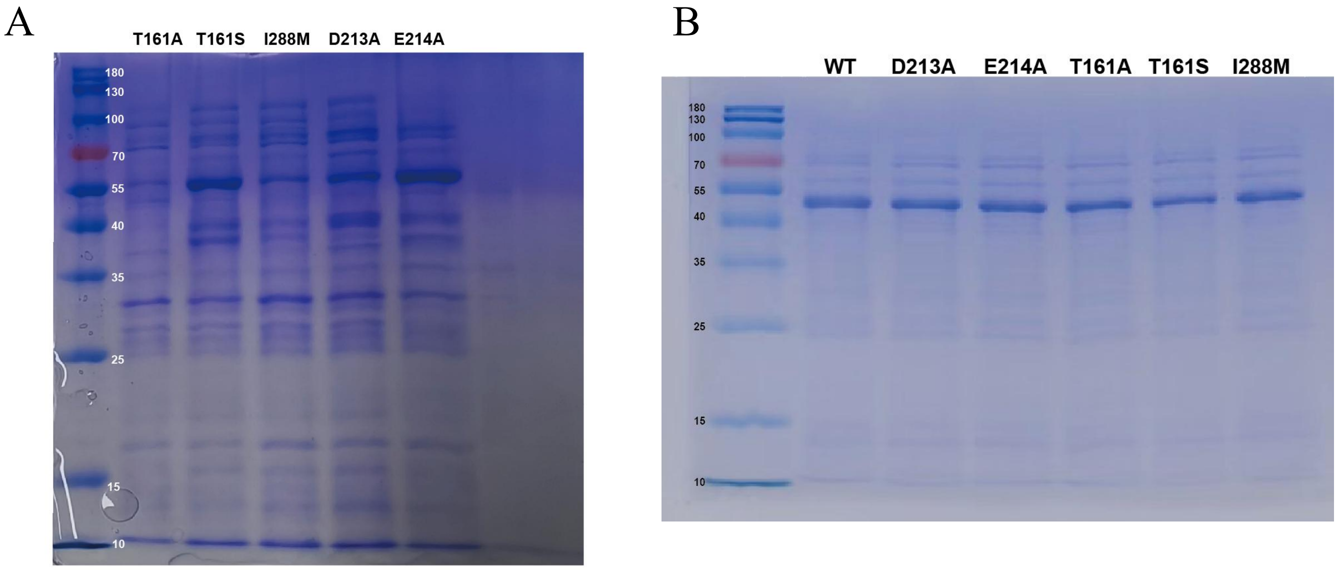

Supplementary Figure S4. Purification analysis of recombinant PfVPS4 mutants

A: SDS-PAGE analysis of five PfVPS4 point mutants following initial purification by Ni-NTA affinity chromatography. **B:** SDS-PAGE verification of the purified mutants after additional gel filtration chromatography. All protein samples were normalized to uniform concentration by BCA assay prior to electrophoresis.

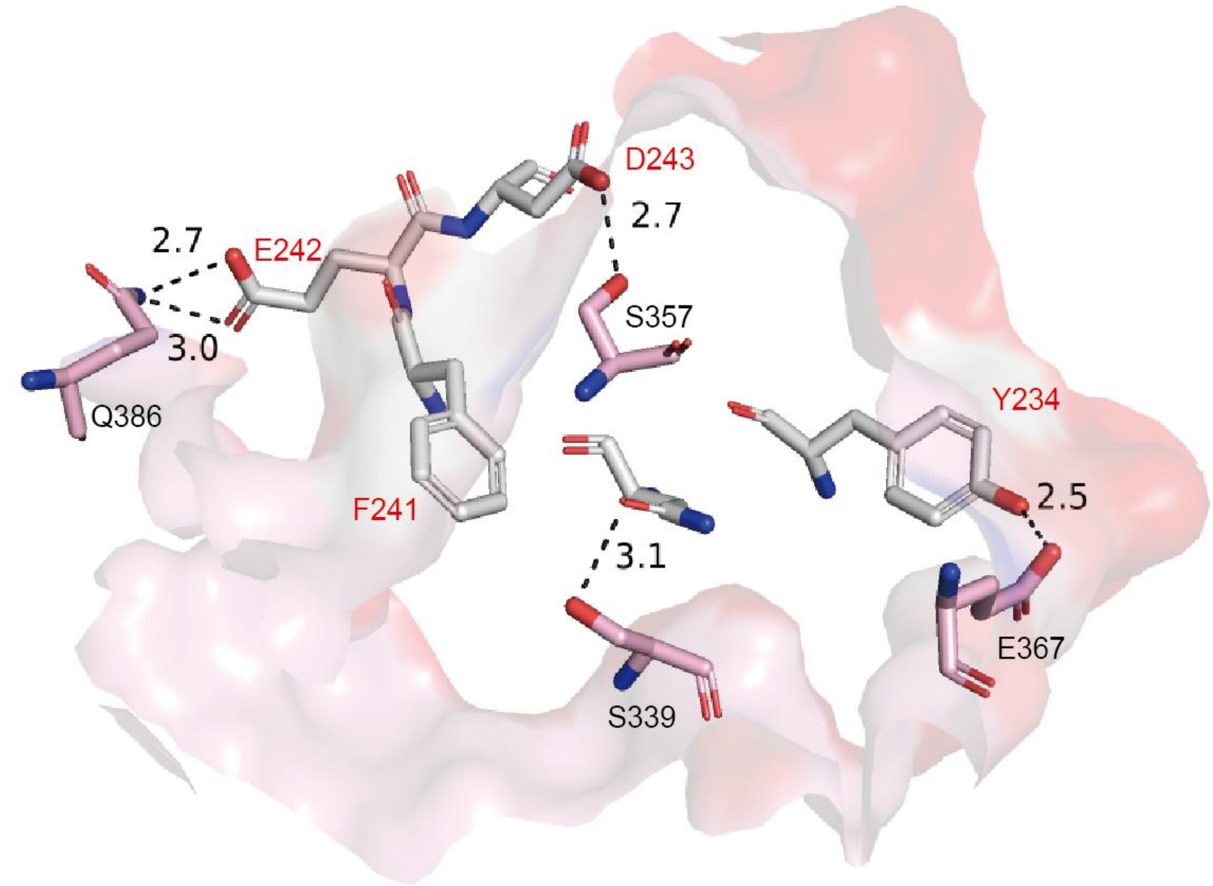

S5 Fig. Structural analysis of the PfVTA1-PfVPS4 interaction interface.

Surface representation of key amino acid residues at the PfVTA1-PfVPS4 binding interface identified in Fig 3A. PfVTA1 is colored red, with residues Y234 and F241 indicated; PfVPS4 is shown in pink. The surface models demonstrate the complementary interface architecture between the two proteins.
